# 30 Jahre prophylaktische Thyreoidektomie beim hereditären medullären Schilddrüsenkarzinom: Ein Meilenstein translationaler Medizin

**DOI:** 10.1007/s00104-024-02105-x

**Published:** 2024-05-28

**Authors:** Henning Dralle, Frank Weber, Kerstin Lorenz, Andreas Machens

**Affiliations:** 1 Klinik für Allgemein‑, Viszeral-und Transplantationschirurgie, Sektion Endokrine Chirurgie, Universitätsmedizin Essen, Hufelandstr. 55, 45147 Essen, Deutschland; 2 Klinik für Viszeral‑, Gefäss- und Endokrine Chirurgie, Universitätsmedizin Halle, Ernst Grube Str. 40, 06097 Halle (Saale), Deutschland

**Keywords:** Multiple endokrine Neoplasie Typ 2, Prophylaktische Thyreoidektomie, RET-Protoonkogen, Kalzitonin, Personalisierte Medizin, Multiple endocrine neoplasia type 2, Prophylactic thyroidectomy, RET protooncogene, Calcitonin, Personalized medicine

## Abstract

Das medulläre Schilddrüsenkarzinom (MTC) ist die häufigste das onkologische Outcome bestimmende Manifestation der multiplen endokrinen Neoplasie (MEN) Typ 2. Vor 30 Jahren konnten die Keimbahnmutationen im *RET(REarranged-during-Transfection)-*Protoonkogen, einem Tumorsuppressorgen auf Chromosom 10q11.2, als Ursache der MEN2 identifiziert und 1993 und 1994 erstveröffentlicht werden. Hieraus entwickelte sich das Konzept der prophylaktischen Thyreoidektomie für asymptomatische Genmutationsträger, das seither Therapiestandard ist. Klinisch-genetische Untersuchungen zeigten hinsichtlich der individuellen Genmutation eine Genotyp-Phänotyp-Korrelation sowohl hinsichtlich der Penetranz und des Entstehungszeitraums des MTC und in geringerem Ausmaß auch hinsichtlich der anderen MEN2-Komponenten Phäochromozytom und primärer Hyperparathyreoidismus. Daraus konnte eine klinisch relevante Risikostratifizierung abgeleitet werden. Die allein genotypbasierte, aber nicht hinreichend genaue Altersempfehlung für den besten Zeitpunkt der prophylaktischen Thyreoidektomie wurde in der Folgezeit durch Kombination des *RET*-Genotyps mit dem Kalzitoninwert präzisiert, der mutations- und altersunabhängig erst bei Überschreiten des oberen Kalzitoninnormwertes das Risiko einer Lymphknotenmetastasierung anzeigt. Die routinemäßige Kalzitoninbestimmung bei Knotenstrumen, das Familienscreening bei MEN2-Indexpatienten und die karzinompräventive prophylaktische Thyreoidektomie bei normokalzitoninämischen Genmutationsträgern haben dazu geführt, dass heute, 30 Jahre nach der Erstbeschreibung der krankheitsverursachenden Genmutationen, das lebensbedrohende hereditäre MTC heilbar geworden ist: ein leuchtendes Beispiel für den Erfolg translational transnationaler medizinischer Forschung zum Wohl der Betroffenen.

## Abstract (en)

Medullary thyroid cancer (MTC) is the most frequent manifestation of multiple endocrine neoplasia type 2 (MEN2) that determines the oncological outcome. Germline mutations in the *rearranged during transfection *(*RET*) protooncogene, a tumor suppressor gene on chromosome 10q11.2, were identified 30 years ago as the genetic basis of MEN2 and published in 1993 and 1994. These seminal findings gave rise to the concept of prophylactic thyroidectomy for asymptomatic gene mutation carriers based on a positive *RET* gene test, which has become the standard of care ever since. Clinical genetic investigations showed genotype-phenotype correlations with respect to the individual gene mutation regarding the penetrance and onset of MTC and to a lesser extent also with respect to the other components of MEN2, pheochromocytoma and primary hyperparathyroidism. From this a clinically relevant risk stratification could be derived. Initially, the optimal timing of prophylactic thyroidectomy was primarily based on the *RET* genotype alone, which was not sufficient for a precise age recommendation and subsequently required additional consideration of calcitonin serum levels for fine tuning. Calcitonin levels first show the risk of lymph node metastasis when they exceed the upper normal limit of the assay independent of carrier age and *RET* mutation. Routine calcitonin screening of patients with nodular thyroid disease, screening of families on identification of MEN2 index patients, and pre-emptive thyroidectomy in carriers of gene mutations with normal calcitonin levels have led to the fact that nowadays, 30 years after the first description of the gene mutations causing the disease, the life-threatening hereditary MTC has become curable: a shining example for the success of translational transnational medical research for the benefit of patients.

In den Jahren 1993 und 1994 beschrieben unabhängig voneinander drei Arbeitsgruppen die genetische Grundlage der zum hereditären medullären Schilddrüsenkarzinom (MTC) führenden multiplen endokrinen Neoplasie Typ 2 (MEN2; [1–3]). Bei diesen über 50 krankheitsverursachenden Mutationen handelt es sich in den meisten Fällen um heterozygot (Mutierung eines der beiden Allele) vorliegende Keimbahnmutationen im *RET*(*REarranged-during-Transfection*)-Protoonkogen, einem Tumorsuppressorgen auf Chromosom 10q11.2. Diese Keimbahnmutationen führen, zumeist unter Austausch einer Aminosäure für eine andere Aminosäure (sog. Missense-Mutationen), in Abwesenheit von Substrat zu einer konstitutiven Aktivierung des membrangebundenen RET-Tyrosinkinase-Rezeptors, der vom mutierten *RET*-Gen kodiert wird. Der RET-Rezeptor wird vor allem in den C‑Zellen der Schilddrüse, den chromaffinen Zellen des Nebennierenmarks und in den Hauptzellen der Nebenschilddrüse exprimiert. Auf diese Weise bestimmen aktivierende, sog. „Gain-of-function“-Mutationen Penetranz und Entstehungszeitraum des MTC und in geringerem Ausmaß auch der beiden anderen endokrinen Syndromkomponenten Phäochromozytom (PCC) und primärer Hyperparathyreoidismus (pHPT). Die aggressivste Form des sich bereits im 1. Lebensjahr entwickelnden hereditären MTC wird durch eine Keimzellmutation des Codon 918 des *RET*-Gens (p.Met918Thr) verursacht. Hierbei entwickelt sich im Gegensatz zum MEN2A-Syndrom kein pHPT, jedoch verschiedene nichtendokrine Stigmata der Augen, des Zungen/Mundbereiches, Intestinaltraktes und Skeletts, sodass dieses Syndrom als MEN2B von der MEN2A genetisch und klinisch getrennt wird.

Im Jahr 1994 wurde kurze Zeit nach Publikation der krankheitsverursachenden Keimzellmutationen, zuerst in den USA [4], dann auch in den Niederlanden [5] und den deutschsprachigen Ländern ([6–8]; Abb. 1), das Konzept der genotypbasierten prophylaktischen Thyreoidektomie etabliert. Die „molekulargenetische Ära“ löste damit die zunächst rein „pathomorphologische“, dann die „biochemische Ära“ [9] ab, in der sich blutsverwandte Mitglieder von MEN2A-Familien wiederholten Kalzitoninuntersuchungen unterziehen mussten, um bei erhöhtem Kalzitonin die Indikation zur Thyreoidektomie stellen zu können. Die damit verbundene individuelle Belastung vor allem derjenigen Familienmitglieder, bei denen keine Keimbahnmutation des *RET*-Protoonkogens vorlag, fand damit ein Ende. Auch falsch-positive oder -negative Kalzitoninergebnisse trugen zu den insgesamt unbefriedigenden Heilungsraten des biochemischen Screenings der vormolekulargenetischen Ära bei MEN2-Familien bei.

**Abb. 1:**
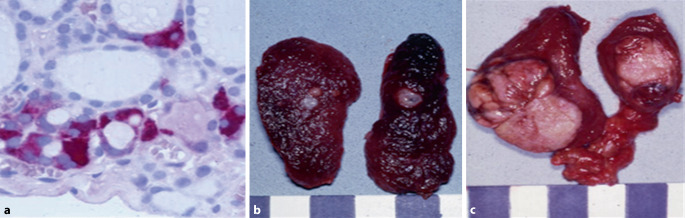
Neoplastische C‑Zell-Hyperplasie (**a**), hereditäres medulläres Mikrokarzinom (**b**) und hereditäres medulläres Makrokarzinom (**c**). **a** Immunhistochemie der ersten DNA-basierten prophylaktischen Thyreoidektomie des Erstautors am 15.09.1994 bei einem 6‑jährigen Jungen mit Mutation im Codon 634

Rückblickend sind 30 Jahre von über 200 Jahren der wissenschaftlichen Erforschung des MTC eine relativ kurze Zeitspanne. Die entscheidenden klinisch-onkologischen Fortschritte beruhen jedoch auf diesen 30 Jahren. Das hereditäre MTC ist damit ein leuchtendes Beispiel für den Erfolg translationaler Forschung in der Medizin. Erst durch sie wurde das hereditäre MTC, ein zuvor nicht selten letal verlaufendes, jedenfalls oft nicht mehr kurativ behandelbares Malignom, zu einem chirurgisch durch prophylaktische Thyreoidektomie heilbaren Tumor.

## Das pathomorphologische Konzept

Wann das MTC als histopathologische Besonderheit erstmals beschrieben wurde, ist unklar. Richard B. Welbourne zitiert in seinem Buch *The History of Endocrine Surgery *[10] Allan Burns aus Glasgow, der 1811 ausführlich von einem langsam wachsenden, unregelmäßig begrenzten, steinharten „medullary sarcoma“, ein Fallbericht von John Abernethy, aus London berichtete [11]. Im Gegensatz zu den papillären und Adenokarzinomen der Schilddrüse, die in der zweiten Hälfte des 19. und ersten Hälfte des 20. Jahrhunderts im Mittelpunkt des Interesses von Pathologen und Chirurgen standen [12–14], führten „medulläre“ Tumoren eher ein Schattendasein. Sie wurden teils den Sarkomen, teils den undifferenzierten Tumoren zugeordnet. Wegen ihres in dieser Zeit meist fortgeschrittenen, nur noch palliativ therapierbaren Stadiums standen sie außerhalb des klinischen und wissenschaftlichen Fokus. So verwundert es daher auch nicht, dass erst 1901 der erste Fall eines mutmaßlichen MEN2B-Patienten von Walther Burk in Tübingen in seiner Dissertation als autoptischer Fall beschrieben wurde [15] – noch in Unkenntnis des histopathologischen Ursprungs und der syndromatischen Zusammenhänge. Der von Burk beschriebene 11-jährige Junge wies MEN2B-typische Stigmata mit „schmächtiger“ Gestalt, „wulstigen“ Lippen, und einem schlanken Thorax mit spitzem epigastrischem Winkel auf, während sein amyloidhaltiger maligner Tumor lokal fortgeschritten war und Fernmetastasen gesetzt hatte.

Der pathohistologische Begriff „medulläres Karzinom“ und seine Definition als eigenständige Entität unter den verschiedenen Tumortypen der Schilddrüsenkarzinome wurde 1959 von Hazard, Hawk und Crile eingeführt und begründet [16]. Die Bezeichnung „Medullarkrebs“ war bereits Ende des 19. und Anfang des 20. Jahrhunderts eine geläufige Bezeichnung für das „solide“ Schilddrüsenkarzinom gewesen, wobei allerdings die Abgrenzung zu undifferenzierten und extensiv wachsenden „Adenokarzinomen“ noch nicht klar definiert war [17]. Hinsichtlich der unterschiedlichen Ausformungen medullärer Karzinome sind an der Beschreibung Wegelins in Bezug auf neueste Erkenntnisse folgende Beobachtungen besonders interessant:

„Bei gleichem Charakter der Epithelzellen können durch die wechselnde Menge des Stromas recht verschiedene Bilder entstehen und die Extreme sind hier wie in anderen Organen der Medullarkrebs und der Szirrhus.“

Und weiter:

„In der Regel nimmt der Tumor einen Schilddrüsenlappen ganz oder teilweise ein, … doch kommt ausnahmsweise auch eine multizentrische Entstehung vor, z. B. hat Langhans in einer Schilddrüse 5 Krebsknoten gesehen, die alle dasselbe makro- und mikroskopische Aussehen zeigten und sogar gegen die Umgebung schön abgekapselt waren“ [17].

Projiziert auf die heutige Erkenntnislage ist bemerkenswert, dass die Pathologie schon vor über 100 Jahren die typischen Merkmale und Formen des medullären Schilddrüsenkarzinoms richtig beschrieben hatte: die Besonderheit des Stromas, heute der Desmoplasie, und das Vorkommen multifokaler Tumoren, heute der Heredität [18–24].

Die erstmalige, weltweit anerkannte Einordnung des medullären Karzinoms erfolgte 1974 in der 1. Auflage der WHO-Klassifikation der Schilddrüsentumoren durch Hedinger und Sobin [25], zuletzt aktualisiert in der 5. Auflage 2022 [26]. Die erst später als Ursprungszelle medullärer Schilddrüsenkarzinome erkannten C‑Zellen wurden vermutlich erstmals von E. Cresswell Baber beim Hund als zwischen den Schilddrüsenfollikeln liegende Zellen beschrieben [27]. In menschlichen Schilddrüsen liegen jedoch mehr als 99 % der C‑Zellen innerhalb, nicht außerhalb der Schilddrüsenfollikel, sodass die Bezeichnung als „parafollikuläre“ Zellen nicht zutreffend ist [21]. Die Bezeichnung „C-Zelle“ geht auf Copp und Cameron (1961) zurück [28], die ihre aus Gewebeextrakten von Hunden gewonnenen Zellen allerdings fälschlicherweise den Nebenschilddrüsen zuordneten. Erst Foster et al. (1964) konnten nachweisen, dass das Kalzitonin der Schilddrüse entstammt [29]. E.D. Williams zeigte dann erstmals, dass die C‑Zellen das morphologische Substrat der medullären Karzinome sind [30].

Der epitheliale Pol der Schilddrüse besteht zu über 99,9 % aus Follikelzellen und nur zu maximal 0,1 % aus C‑Zellen [21]. Angesichts dieses geringen Anteils an C‑Zellen erscheint der Anteil medullärer Karzinome von ca. 5 % unter den Schilddrüsenkarzinomen mindestens 50fach überrepräsentiert. Eine Erklärung gibt es hierfür bislang nicht. C‑Zellen sind über die gesamte Schilddrüse, mit Ausnahme des Schilddrüsenisthmus und Lobus pyramidalis, verteilt, bevorzugen jedoch die zentralen und laterodorsokranialen Anteilen der Schilddrüse. Dementsprechend entstehen medulläre Karzinome in diesen Bereichen am häufigsten. Da diese bevorzugten Schilddrüsenareale lymphogen entlang der oberen Schilddrüsenvene nach lateral drainieren, treten laterale Lymphknotenmetastasen von Tumoren dieser Schilddrüsenanteile als sog. „Skip-Metastasen“ – unter Umgehung der zentralen Halslymphknoten, die scheinbar „übersprungen“ werden – in bis zu 20 % der Fälle in Erscheinung [31–33]. Es überrascht andererseits aber auch nicht, dass medulläre Karzinome im Schilddrüsenisthmus oder Lobus pyramidalis eine absolute Rarität sind.

**Abb. 2:**
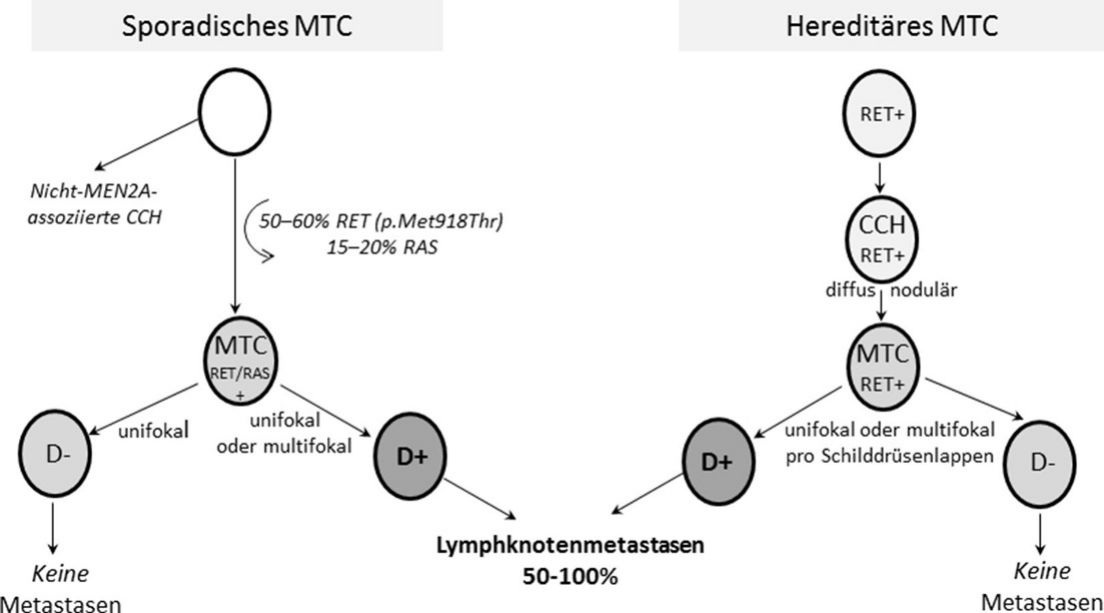
Das pathologische Konzept: Histopathogenese des sporadischen und hereditären MTC. *CCH* C-Zell-Hyperplasie, *D−/D+* Desmoplasie negativ/positiv, *MTC* medulläres Schilddrüsenkarzinom; *RET* *REarranged-during-Transfection*-Protoonkogen

Sporadische MTC entstehen durch somatische, sich wechselseitig ausschließende *RET* (50–60 %) und *RAS* (15–20 %) -Mutationen in einzelnen C‑Zellen [34], während beim hereditären MTC in allen Körperzellen *RET*-Mutationen vorliegen. Multifokalität kommt bei sporadischen MTC nur bei desmoplasiepositiven Primärtumoren vor [21], stellt bei hereditären MTC hingegen einen krankheitstypischen Befund dar. Sporadische MTC entstehen immer direkt aus der mutierten C‑Zelle. Hereditäre MTC führen erst über Zwischenstufen neoplastischer C‑Zell-Hyperplasien (CCH) zum MTC, wenn die nodulär wachsenden C‑Zellen die Basalmembran überschritten haben ([35–37]; Abb. 1, 2).

Eine neoplastische CCH findet sich regelhaft in Präparaten von Patienten mit Disposition zum hereditären MTC infolge einer *RET*-Keimbahnmutation. Sie ist mit ihren charakteristischen Merkmalen bereits im HE-Schnitt erkennbar [21]. Die nichthereditäre, nichtneoplastische, beim Nachweis von mindestens 50 benachbarten C‑Zellen zu diagnostizierende CCH ist dagegen nur mit der Kalzitoninimmunhistochemie nachweisbar. Sie wird bei verschiedenen benignen und malignen nicht-MTC-assoziierten Pathologien beobachtet und ist daher morphologisch und klinisch von der neoplastischen, keimzellmutationassoziierten CCH streng zu unterschieden. Die nichtneoplastische CCH ist im Gegensatz zur neoplastischen keimzellmutationassoziierten CCH keine prämaligne Zwischen- oder Vorstufe des sporadischen MTC.

Amyloidablagerungen finden sich in 60–85 % der MTC [21]. Schon in der Dissertation von Walther Burk [15] waren sie die zur weiteren detaillierten Untersuchung führende Auffälligkeit. Da sich Amyloid jedoch auch bei nicht-C-Zell-assoziierten Pathologien der Schilddrüse findet [17, 21, 35] und im Gegensatz zu Desmoplasieherden keine Chromogranin-A-Positivität aufweist [36], kann sich die Diagnose eines MTC nicht allein auf dessen Nachweis stützen [21]. Ganz anders verhält es sich mit der schon von Wegelin sehr ausführlich beschriebenen Stromabildung beim MTC [17]. Nach heutigen Erkenntnissen besteht bei einem desmoplasiepositiven Stroma ein signifikant erhöhtes Metastasierungsrisiko. Umgekehrt wurden bei desmoplasienegativen sporadischen und auch hereditären MTC bislang keine Metastasen nachgewiesen [19, 20, 22–24]. Da der Desmoplasienachweis bereits im Gefrierschnitt geführt werden kann, kommt dem Nachweis bzw. Fehlen von Desmoplasie in medullären Karzinomen ein hinsichtlich des Ausmaßes der Operation entscheidender Stellenwert zu [22–24].

Bevor Walther Burk 1901 den wahrscheinlich ersten Fall eines hereditären MEN2B-MTC beschrieb, berichtete Felix Fränkel 1886 über den ersten Fall eines damals noch unbekannten MEN2A-Syndroms bei einer obduzierten 18-jährigenFrau mit bilateralen Nebennierentumoren und Struma [37]. 120 Jahre später gelang es an noch von diesem spektakulären Fall erhaltenen Material die krankheitsauslösende Mutation im *RET*-Protoonkogen Codon 634 (p.Cys634Trp) nachzuweisen und damit zweifelsfrei zu belegen, dass es sich bei dem 1886 von Fränkel publizierten Fall um ein hereditäres MEN2A-Syndrom handelte [38]. Erst 1968 beschrieben Steiner und Mitarbeiter anhand einer Familie mit MTC, PCC und pHPT erstmals die klassischen Charakteristika des von den Autoren so benannten MEN2A-Syndroms [39]. Teilaspekte des MEN2A-Syndroms waren bereits von anderen Autoren erst wenige Jahre zuvor publiziert worden [40–43]. Allerdings wurde bei dem nach dem Erstbeschreiber Sipple [44] bisweilen auch als „Sipple-Syndrom“ bezeichneten MEN2A-Syndrom nicht ein medulläres, sondern ein follikuläres Schilddrüsenkarzinom [10] beschrieben.

Zusammenfassend stellt der histologische Nachweis einer neoplastischen CCH meist mit, aber auch ohne uni- oder multifokale MTC das pathomorphologische Substrat der Keimzellmutation des *RET*-Protoonkogens und hereditären MTC dar. Der histologische Befund sollte daher stets Ausgangspunkt einer individuellen Tumordiagnostik bez. PCC und pHPT sein und im Falle eines Indexpatienten ein Familienscreening zur Folge haben.

## Das biochemische Konzept

Kalzitonin ist in mehrfacher Hinsicht ein einzigartiger, hochspezifischer Tumormarker des MTC:

- Es gibt nur in extrem seltenen Fällen extrathyreoidal neuroendokrine Karzinome mit Kalzitoninsekretion [45–48].
- Nichtkalzitoninsezernierende MTC sind mit 0,8 % außerordentlich selten [49].
- Die Höhe der Kalzitoninwerte korreliert mit der Tumorzellmasse und mit der Tumorzelldifferenzierung sowohl präoperativ als auch bei persistierenden oder rezidivierenden MTC im Verlauf nach Operation [33, 50–53].
- Die Kalzitoninverdopplungszeit hat im Rahmen der Tumornachsorge prognostische und therapeutische Bedeutung [54].
- Bei MEN2A-Genmutationsträgern entscheidet die Höhe der Kalzitoninwerte über den spätestmöglichen Zeitpunkt der kurativen prophylaktischen Thyreoidektomie [55].

Demgegenüber sind bei der Interpretation der Kalzitoninwerte, von Assay-spezifischen Besonderheiten [56] abgesehen, nur wenige Punkte zu berücksichtigen. Gering erhöhte Kalzitoninwerte bei benignen Schilddrüsenerkrankungen sind zumeist Ausdruck einer nichtneoplastischen, nicht zum MTC prädisponierenden, sporadischen CCH [57]. Unter Beachtung der geschlechtsspezifischen Unterschiede der Kalzitoninwerte [58] wird daher seitens der Sektion Schilddrüse der Deutschen Gesellschaft für Endokrinologie empfohlen, basale Kalzitoninwerte über 30 pg/ml bei Frauen bzw. über 60 pg/ml bei Männern als Operationsindikation mit Verdacht auf sporadisches MTC zu bewerten bzw. im Zweifelsfall nach 3 bis 6 Monaten zu kontrollieren [59, 60]. Zusätzlich zur Geschlechtsspezifik der Kalzitoninwerte besteht darüber hinaus auch eine Altersspezifik. Bei Kindern bis zu 3 Jahren, insbesondere im 1. Lebenshalbjahr, sind die Kalzitoninwerte deutlich höher (15–40 pg/ml) als bei Kindern über 3 Jahren und im Jugend- und Erwachsenenalter [61].

Die Interpretation der Kalzitoninwerte von Patientinnen und Patienten mit sporadischen und hereditären MTC und prä- bzw. postoperativ erhöhten Kalzitoninwerten lässt keine Differenzierung lokoregionärer Lymphknoten bzw. Fernmetastasen zu. Nicht selten können insbesondere postoperativ bei signifikanter Kalzitoninerhöhung trotz moderner Bildgebung keine Metastasen nachgewiesen werden, wenngleich bei Kalzitoninwerten über 500–1000 pg/ml Mikro- bzw. Makrometastasen in anderen Organen höchstwahrscheinlich sind [33, 62].

**Abb. 3:**
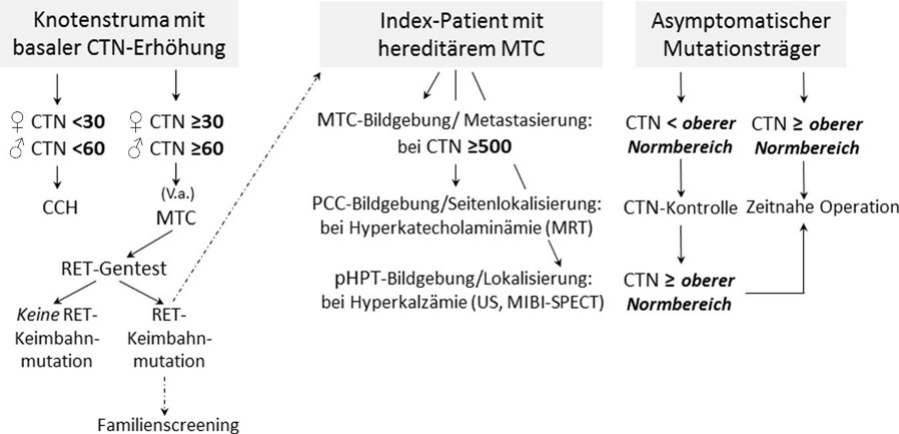
Das biochemische Konzept: Evaluation des sporadischen und hereditären MTC mittels Serumkalzitoninbestimmung. CTN-Werte sind in pg/ml angegeben. *CCH* C-Zell-Hyperplasie, *CTN* basales Serumkalzitonin, *MIBI-SPECT* Nebenschilddrüsenszintigraphie, *MTC* medulläres Schilddrüsenkarzinom, *PCC* Phäochromozytom, *pHPT* primärer Hyperparathyreoidismus, *RET* REarranged-during-Transfection-Protoonkogen, *US* Ultraschall

Das biochemische Konzept beim hereditären MTC verfolgt folgende Hauptziele (Abb. 3):

1. *Frühdiagnose des MTC durch routinemäßige Bestimmung des basalen Kalzitonins bei benignen Schilddrüsenerkrankungen als Bestandteil der krankheitsbezogenen In-vitro-Diagnostik*

Innerhalb dieser Patientenpopulation hängt die Wahrscheinlichkeit der Frühdiagnose eines MTC von den individuellen Befunden, insbesondere der Höhe des als pathologisch definierten Kalzitoningrenzwertes ab. Bei Frauen mit einem Kalzitoninwert über 30 pg/ml bzw. bei Männern über 60 pg/ml liegt höchstwahrscheinlich ein MTC vor [60]. Bei ca. 25 % aller Patientinnen und Patienten mit MTC findet sich eine Keimbahnmutation im *RET*-Protoonkogen. Bis zu 10 % dieser De-novo-Keimbahnmutationen sind in den Keimzellen phänotypisch gesunder Eltern, insbesondere des Vaters aufgrund der größeren Zahl von Zellteilungen bei der Spermatogenese [63–65], entstanden oder betreffen neu diagnostizierte solitäre MTC, die bei negativer Familienanamnese primär als sporadisch eingeschätzt wurden [66]. In Deutschland besteht daher im Gegensatz zu den USA [67] Konsens in der Empfehlung zur routinemäßigen Kalzitoninbestimmung bei Patientinnen und Patienten mit Knotenstrumen [59, 60], um medulläre Karzinome zunächst unabhängig von ihrer genetischen Disposition frühzeitig zu diagnostizieren und beim Nachweis einer *RET*-Mutation entsprechend chirurgisch zu entfernen. Beim hereditären MTC ist eine totale Thyreoidektomie immer erforderlich, beim sporadischen MTC nur dann, wenn ein desmoplasiepositives MTC oder zusätzlich eine bilaterale Knotenstruma vorliegt [23]. Die initiale Kalzitoninbestimmung mit nachfolgender *RET*-Genanalyse ist daher nicht nur für die frühzeitige Erkennung von Indexpatienten wichtig, sie hat auch eine wesentliche Bedeutung für die Planung des Operationsausmaßes.

2. *Syndromspezifische Diagnostik bez. PCC und pHPT beim Indexpatienten eines hereditären MTC und Einleitung eines Familienscreenings*

Im Vergleich zu asymptomatischen, durch Familienscreening diagnostizierten Genmutationsträgern sind Indexpatienten meist älter, haben größere Primärtumoren, wesentlich häufiger Lymphknotenmetastasen und somit deutlich geringere Chancen auf eine biochemische Heilung nach der Operation (Tab. 1). Zur Planung und Vorbereitung der Operation beim Indexpatienten sollte eine biochemische und ggf. bildgebende Diagnostik zum Nachweis bzw. Ausschluss eines PCC bzw. pHPT durchgeführt werden. Während bei asymptomatischen Genmutationsträgern die Synchronmanifestation von MTC und PCC extrem unwahrscheinlich ist, kann dies bei Indexpatienten in entsprechendem Alter durchaus vorkommen [68–70]. Im positiven Fall einer Synchronmanifestation ist operationsvorbereitend zu klären, ob eine uni- oder bilaterale PCC-Erkrankung vorliegt. Bei Indexpatienten mit Kalzitoninwerten über 500 pg/ml empfiehlt sich darüber hinaus zum lokoregionären und distanten Staging eine entsprechende Bildgebung.

3. *Kalzitoninbestimmung zur Evaluation des optimalen Zeitpunkts der prophylaktischen Thyreoidektomie bei asymptomatischen Genmutationsträgern einer hereditären C‑Zell-Erkrankung *[55, 71]

**Tab. 1:** Hereditäres MTC: pathologische und klinische Befunde bei Screening- und Indexpatienten mit MEN2A-Syndrom. (Mod. nach [70])

	Screeningpatienten^a^	Indexpatienten	*p*
*Geschlecht (weiblich, %)*	55,2	60,9	0,224
*Mittleres Alter bei MTC-Operation (Jahre)*	30,5	45,5	< 0,001
Risikogruppe „high“^b^ (Jahre)	22,4	33,7	0,002
Risikogruppe „moderate high“ (Jahre)	36,1	41,9	
Risikogruppe „low moderate“ (Jahre)	41,4	55,3	
*Mittlerer Durchmesser größter Primärtumor (mm)*	7,9	19,5	< 0,001
Risikogruppe „high“ (mm)	8,0	22,4	< 0,001
Risikogruppe „moderate high“ (mm)	10,6	23,2	< 0,001
Risikogruppe „low moderate“ (mm)	5,4	16,0	< 0,001
*pN1->MTC (%)*	29,5	71,5	< 0,001
*Biochemische Heilung MTC*^*c*^	74,8	34,1	< 0,001
*Phäochromozytomoperation*			
Patienten (%)	13,2	30,1	< 0,001
Mittleres Alter (Jahre)	35,7	37,5	0,431
*pHPT-Operation*			
Patienten (%)	17 (3,8)	14 (9,0)	0,019
Mittleres Alter (Jahre)	36,4	40,9	0,349

^a^Alle sowohl prophylaktisch als auch nichtprophylaktisch operierten Patienten, die im Rahmen des Familienscreening als *RET*-Mutation-positive Genträger identifiziert wurden

^b^Risikogruppe „high“: *RET*-Mutation Codon 634; „moderate high“: *RET*-Mutationen Codon 609, 611, 618, 620, 630; „low moderate“: *RET*-Mutationen 768, 790, 804, 891

^c^Biochemische Heilung: postoperativ im Langzeitverlauf Normokalzitoninämie

Während anfänglich Stimulationstests mit Pentagastrin [52] und später, als Pentagastrin nicht mehr erhältlich war, mit Kalzium zur Ersteinschätzung des MTC-Risikos verwendet wurden [57], zeigte sich, dass hierfür die alleinige Bestimmung des basalen Kalzitonins aufgrund der hohen Sensitivität moderner Kalzitonin-Assays ausreichend sicher ist. Solange die basale Serumkalzitoninkonzentration im Referenzbereich liegt, kann davon ausgegangen werden, dass eine CCH (78 %) oder ein MTC (22 %) vorliegt und noch keine Lymphknoten- oder Fernmetastasierung stattgefunden hat [55]. Mit diesen von zwei unabhängigen Arbeitsgruppen erzielten Ergebnissen [55, 71] konnte auf die belastenden Stimulationstests verzichtet werden. Dies stellt eine Erleichterung für die betroffenen Mutationsträger dar, die auch ohne Stimulationstest weiterhin sicher sein können, bei regelmäßigem biochemischem Monitoring den letztmöglichen Zeitpunkt für eine kurative Thyreoidektomie nicht zu verpassen.

Solange sich der Kalzitoninwert im Referenzbereich befindet und die Notwendigkeit einer zeitnahen Thyreoidektomie bei Genmutationsträgern noch nicht besteht, sollten die Intervalle der Kalzitoninbestimmung vom basalen Kalzitoninausgangswert und der Risikogruppe der individuell vorliegenden *RET*-Mutation (Tab. 1) abhängig gemacht werden. Beim Vorliegen der Hochrisikogruppe mit Mutation im Codon 634 des *RET*-Protoonkogens sollten die Intervalle kürzer sein, z. B. halbjährlich, als bei den anderen Risikogruppen, bei denen z. B. ein- bis zweijährlich ausreichend erscheint. In jedem Fall besteht, solange der Kalzitoninwert im Normbereich liegt, die individuelle Option einer „frühzeitig prophylaktischen“ Operation, z. B. vor der Einschulung des Kindes, vor der Pubertät oder danach, oder einer Operation erst zu einem „Last-minute“-Zeitpunkt, wenn sich das Kalzitonin dem oberen Grenzbereich nähert.

Ausgenommen von dieser am individuellen Kalzitoninwert ausgerichteten Strategie sind in jedem Fall Träger einer MEN2B-Mutation im Codon 918 (p.Met918Thr), die so früh als möglich, d. h. bestenfalls im 1. Lebensjahr operiert werden sollten [72–74]. Bei dieser aggressivsten Form des MEN2-Syndroms kommt es bereits im 1. Lebensjahr zur Transformation der CCH zum MTC mit sehr frühzeitiger, nur noch im Ausnahmefall kurativ behandelbarer Lymphknotenmetastasierung. Kinder von MEN2B-Eltern sollten daher bereits nach der Geburt gengetestet werden, um eine Thyreoidektomie schon im 6. bis 12. Lebensmonat zu ermöglichen [74]. Bei „de novo“ erkrankten MEN2B-Kindern ist außerordentlich wichtig, dass Kinderärzte die speziellen, z. T. sich unmittelbar nach der Geburt entwickelnden nichtendokrinen Frühsymptome einer MEN2B-Ersterkrankung zeitnah erkennen und durch Kalzitoninbestimmung und Gentestung weiter abklären.

Charakteristische, bei fast allen Kindern mit MEN2B-Syndrom feststellbare Frühsymptome sind tränenloses Weinen und Obstipation [75, 76]. Obwohl das Erscheinungsspektrum und dementsprechend das Alter bei Diagnose der MEN2B-Patienten mit De-novo-Mutation von bis zu 34 Jahren sehr breit ist [72], kann bei einer Operation jenseits des 4. Lebensjahrs nicht mehr von einer onkologisch heilbaren Situation ausgegangen werden. Die ausschlaggebende Heilungschance dieser aggressiven C‑Zell-Erkrankung liegt daher im Erkennen und Zuordnen der nichtendokrinen Frühsymptome tränenloses Weinen und Obstipation. Die ansonsten als typisch beschriebenen phänotypologischen Veränderungen wie z. B. wulstige Lippen und Schleimhautneurome der Zunge werden nur selten vor dem 4. Lebensjahr sichtbar, wenn bereits keine Aussichten auf Heilung mehr bestehen.

## Das chirurgische Konzept

**Abb. 4:**
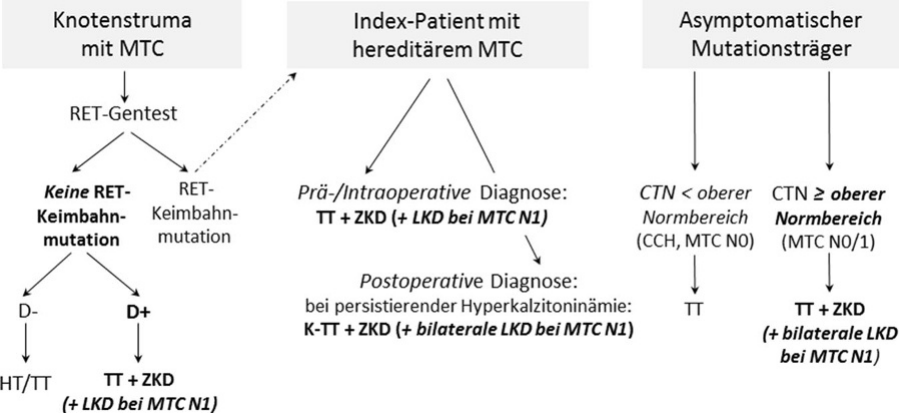
Das chirurgische Konzept: Resektionsausmaß bei Knotenstruma mit MTC, Indexpatienten mit hereditärem MTC und asymptomatischen Genträgern. *CCH* C-Zell-Hyperplasie, *CTN* basales Serumkalzitonin, *D−/D+* Desmoplasie negativ/positiv, *HT* Hemithyreoidektomie, *K‑TT* Kompletierungs-TT, *LKD* laterale Kompartmentdissektion, *MTC N0/1* nodal-negatives/positives medulläres Schilddrüsenkarzinom, *RET* *REarranged-during-Transfection*-Protoonkogen, *TT* totale Thyreoidektomie, *ZKD* zentrale Kompartmentdissektion

In der Allgemein‑/Viszeralchirurgie ist die prophylaktische Thyreoidektomie bei hereditären C‑Zell-Erkrankung das bislang einzige auf einer einzelnen Genmutation beruhende karzinompräventive Konzept. Die Penetranz der MEN2-Erkrankung zu Lebzeiten beträgt selbst bei Trägern von Niedrigrisikomutationen in Abhängigkeit vom erreichten Lebensalter nahezu 100 % für die Erstmanifestation hereditäres MTC. Anders als z. B. beim hereditären, nichtneuroendokrinen Darmkrebs besteht beim hereditären MEN2-MTC eine enge Genotyp-Phänotyp-Korrelation, die nicht nur den Alterskorridor und die transformative Aktivität der C‑Zell-Erkrankung bestimmt, sondern auch Häufigkeit, Manifestationsalter und Rezidivrisiko der syndromassoziierten Phäochromozytom- und Nebenschilddrüsentumoren beeinflusst [77–79]. Hinsichtlich des in diesem Beitrag beschriebenen hereditären MTC unterscheiden sich daher auch die Ziele des operativen Konzepts in Abhängigkeit von der biochemisch und molekulargenetisch untersuchten Zielgruppe bei (a) Patienten mit Knotenstruma, (b) Indexpatienten eines hereditären MTC und (c) asymptomatischen Genmutationsträgern (Abb. 4).

### Patienten mit Knotenstruma

Die auf Grundlage des interdisziplinären Konsenses 2004 empfohlene routinemäßige Kalzitoninbestimmung bei Patienten mit Knotenstruma [59] hat in Verbindung mit der Empfehlung, alle MTC-Patienten unabhängig vom familiären oder individuellen Vorhandensein MEN2-typischer Tumoren genetisch zu testen, dazu geführt, dass sowohl sporadische als auch hereditäre MTC vermehrt und in einem früheren, prognostisch günstigeren Stadium diagnostiziert werden [9, 80]. Bei Patienten mit Knotenstruma, MTC und negativem *RET*-Gentest liegt ein sporadisches MTC vor, das entweder primär (prä-/intraoperative Diagnose) oder sekundär komplettierend (postoperativ persistierende Hyperkalzitoninämie) entsprechend den internationalen Empfehlungen operiert wird [67]. Beim sporadischen, desmoplasienegativen MTC ist nach heutigen Erkenntnissen, sofern keine behandlungsbedürftige bilaterale Knotenstruma zusätzlich vorliegt, keine primäre oder sekundäre totale Thyreoidektomie erforderlich [23]. Die desmoplasienegative medulläre Neoplasie ist eine eigenständige, unifokale, nichtinvasive, nichtmetastasierende Tumorentität, die bei 25–30 % der sporadischen MTC-Patienten nachweisbar ist [23]. Da bei dieser Tumorform, vergleichbar der enkapsulierten nichtinvasiven follikulären Neoplasie mit papillärähnlichen Kernmerkmalen (NIFTP; [81, 82]), bislang keine Metastasierung nachgewiesen wurde [19, 20, 22, 23], ist eine Lymphknotendissektion, anders als beim invasiven, desmoplasiepositiven MTC, wegen der hierbei häufigen Lymphknotenmetastasierung, nicht erforderlich. Wegen der beim desmoplasienegativen sporadischen MTC fehlenden Multifokalität ist aus onkologischer Sicht zudem lediglich eine ipsilaterale Hemithyreoidektomie angezeigt [23].

### Indexpatienten eines hereditären MTC

Knotenstrumapatienten mit *RET*-Mutation-positivem MTC ohne patientenseits oder familiär bekannte MEN2-Erkrankung werden als Indexpatienten bezeichnet. Das Risiko von Lymphknotenmetastasen ist bei Indexpatienten mit 72 % deutlich höher als bei Screeningpatienten mit 30 % (Tab. 1; [70]). Das Resektionsausmaß richtet sich daher in erster Linie danach, ob Lymphknotenmetastasen prä-/intraoperativ oder postoperativ nachgewiesen werden. Bei einem N1-Status sollte zusätzlich zur totalen Thyreoidektomie eine bilateral zentrale und bilateral laterale Kompartmentresektion der Level 2 bis 6 durchgeführt werden [67, 83]. Ob auch beim hereditären MTC auf eine tumorseitige laterale Kompartmentresektion verzichtet werden kann, wenn ipsilateral ein desmoplasienegatives MTC vorliegt, wie initiale Untersuchungen vermuten lassen könnten [24], ist Gegenstand der Diskussion und noch nicht abschließend zu bewerten.

Die zur lokoregionären Tumorausdehnung, insbesondere zur Diagnostik von Halslymphknotenmetastasen geeignetste Bildgebung ist der zervikale Ultraschall, bei Verdacht auf Infiltration der zervikoviszeralen Achse ergänzt durch Kontrastmittel-CT oder MRT. Zur Diagnostik von Fernmetastasen hat sich beim MTC die molekulare Bildgebung mittels PET mit verschiedenen Tracern als am aussagekräftigsten erwiesen. Dabei ist 18F-DOPA das bevorzugte Radiopharmakon, alternativ 68Ga-DOTA, oder, insbesondere bei schlecht differenzierten MTC mit stark erhöhtem CEA-Anteil, die FDG-PET. Bei Lungen- und Knochenmetastasen wird ergänzend eine CT, bei Weichgewebs- und Hirnmetastasen eine MRT durchgeführt [84, 85].

Bei präoperativ diagnostizierten Indexpatienten sollte ferner eine Katecholaminbestimmung zum Ausschluss bzw. Nachweis einer PCC-Erkrankung vorliegen (Abb. 3). Je nach Patientenalter und -zustand sowie Ausmaß der MTC- und PCC-Erkrankungen kann eine Synchronoperation [68, 69] oder ein metachrones Vorgehen mit Erstoperation des PCC und Zweiteingriff des MTC erfolgen. Hinsichtlich des Resektionsausmaßes an der bzw. den Nebennieren hat sich beim MEN2-PCC die uni- oder bilateral subtotale Adrenalektomie durchgesetzt, da diese, so technisch möglich, den Erhalt einer Nebennierenrindenhormonrestsekretion zum Erhalt der Stresskompetenz (adrenokortikale Reserve) ermöglicht [68, 86, 87].

Ursprünglich wurde bez. der Syndromkomponente pHPT von einer Häufigkeit von bis zu 25 % ausgegangen. Aktuelle Untersuchungen ergeben mit einer Gesamthäufigkeit des pHPT beim MEN2A-Syndrom von 7,8 % ein anderes Bild. Selbst bei Hochrisikopatienten mit Mutation im Codon 634 fand sich ein pHPT bei lediglich 15 % der Patienten, bei den Patienten mit Niedriger- und Niedrigmutationen in nur 2,2 % [9]. Beim MEN2B-Syndrom kommt ein pHPT nicht vor. Einhergehend mit der niedrigen Penetranz des MEN2A-assoziierten pHPT finden sich im Gegensatz zum MEN1-Syndrom [88] fast immer Eindrüsenerkrankungen der Nebenschilddrüsen mit geringem Rezidivrisiko durch Zweitdrüsenerkrankung nach Entfernung der betroffenen Nebenschilddrüse. Eine „prophylaktische“ Parathyreoidektomie nicht vergrößerter Nebenschilddrüsen sollte daher beim MEN2A-pHPT vermieden werden.

### Asymptomatische Genmutationsträger

Unter asymptomatischen Genmutationsträgern werden im strengen Sinn nur normokalzitoninämische Patienten mit positivem *RET*-Mutationsstatus ohne Lymphknotenmetastasen verstanden. Im Unterschied dazu besteht die Gesamtgruppe der Screeningpatienten nicht nur aus asymptomatischen Genmutationsträgern, sondern auch aus anderen Genmutationsträgern mit unterschiedlichen Stadien der C‑Zell-Erkrankung: C‑Zell-Hyperplasien ohne und mit nodal-negativem oder bereits nodal-positivem MTC. Insgesamt sind daher für die Gruppe der Screeningpatienten die Heilungsraten mit 75 % zwar erheblich besser als für Indexpatienten mit 34 % (Tab. 1; [70]), erreichen aber nicht 100 % wie die Gruppe der normokalzitoninämischen nodal-negativen Genmutationsträger [55].

Nach Identifizierung eines Indexpatienten ist die frühzeitige Durchführung einer individuell angepassten umfassenden Beratung vorrangiges Ziel, um eine regelmäßige Kontrolle familiärer Genmutationsträger schon im Stadium der Normokalzitoninämie zu ermöglichen. Im Rahmen der Erstaufklärung ist diesen bzw. ihren Eltern die Bedeutung des oberen Kalzitoninreferenzbereiches für die Wahl des Zeitpunktes der prophylaktischen Thyreoidektomie zu erläutern. Die Kalzitoninwerte reflektieren mit hinreichender Sicherheit das pathomorphologische Kontinuum der genetisch determinierten C‑Zell-Progression [89]. Sie lassen zwar nicht erkennen, wann es zur Transformation der neoplastischen CCH zum MTC kommt, zeigen jedoch mit dem oberen Referenzwert an, wann das Risiko von Lymphknotenmetastasen ansteigt und sich die Heilungschancen signifikant verschlechtern [55, 71]. Für die Eltern der betroffenen Kinder besteht daher parallel zum pathomorphologisch-biochemischen Kontinuum der C‑Zell-Erkrankung ein zeitlicher Entscheidungskorridor, der, solange der Kalzitoninspiegel im Normbereich bleibt, genügend Zeit und Überlegung erlaubt, eine individuell kindgerechte Wahl zwischen einer „Last-minute“-Thyreoidektomie beim Erreichen des Kalzitoninoberbereiches oder einer „frühzeitig prophylaktischen“ Thyreoidektomie bei stabilen bzw. nur gering und langsam ansteigenden Kalzitoninwerten zu treffen.

**Abb. 5:**
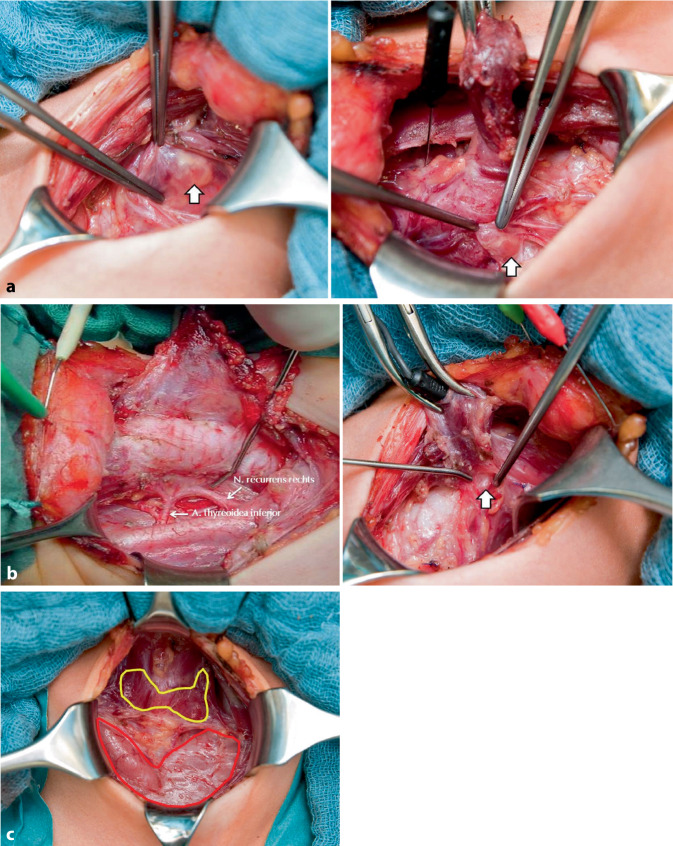
Intraoperative kindliche Anatomie **a** der Schilddrüse und Nebenschilddrüsen (*Pfeil*), **b** der Rekurrensnerven bei MEN2A (*rechts*) und MEN2B (*links*) und **c** des zervikalen Thymus nach Thyreoidektomie bei hereditärer C‑Zell-Erkrankung im Rahmen des MEN2-Syndroms

Die Durchführung der prophylaktischen Thyreoidektomie bei normokalzitoninämischen Patienten hat nicht nur den onkologischen Vorteil einer 100 %igen Heilungschance, sondern macht die Lymphknotendissektion überflüssig, wodurch sich das Risiko eines passageren oder permanenten Hypoparathyreoidismus bzw. einer Stimmlippenparese deutlich reduziert [90, 91]. Wegen der besonderen Anatomie der kindlichen Schilddrüse, Nebenschilddrüsen, Rekurrensnerven und des Thymus (Abb. 5) setzt die Operation insbesondere im frühen Kindesalter eine sehr spezielle operative Erfahrung voraus. Die Nebenschilddrüsen sind nicht nur besonders klein, sondern unterscheiden sich aufgrund ihrer leicht rötlichen Farbe vom Schilddrüsenparenchym schlechter als bei Jugendlichen und Erwachsenen. Die Rekurrensnerven sind beim MEN2A-Syndrom äußerst zart und dünnkalibrig, beim MEN2B-Syndrom dagegen deutlich verdickt und dadurch wesentlich leichter zu identifizieren. Mit geeigneten Techniken können auch im Kleinkindesalter die Vagus-und Rekurrensnerven intermittierend und kontinuierlich stimuliert werden [92]. Die zervikalen Thymi sind im Kindesalter noch sehr prominent und können in den ersten Lebensjahren größer sein als die Schilddrüse (Abb. 5).

## Diskussion

Schon in den Jahren vor der Erstbeschreibung der krankheitsverursachenden Mutationen des *RET*-Protoonkogens war das diagnostische und therapeutische Ziel, Familienmitglieder mit MEN2-Syndrom in einem möglichst frühen Tumorstadium zu erkennen [93]. Die in der vormolekularen Ära zur Früherkennung einzig zur Verfügung stehende Kalzitoninbestimmung hatte jedoch nicht nur Assay-technische Limitationen, sondern auch den wesentlichen Nachteil, im Stadium der Normokalzitoninämie Betroffene nicht sicher von nichtbetroffenen Familienmitgliedern unterscheiden zu können. Damit konnten nicht betroffene Familienmitglieder zunächst nicht als solche erkannt und diesen auch nicht das regelmäßige Kalzitoninmonitoring erspart werden. In der prämolekulären Ära wurden betroffene MEN2-Patienten erst als solche erkannt und einer Thyreoidektomie zugeführt, nachdem der obere Referenzwert des Kalzitonins überschritten worden war und die Heilungsaussichten wegen Lymphknotenmetastasierung deutlich eingeschränkt waren. Die Identifikation der krankheitsverursachenden Mutationen im *RET*-Protoonkogen vor 30 Jahren [1–3] bedeutete daher einen Quantensprung für die Thyreologie, ebenso für die karzinompräventive kurative Chirurgie im Falle einer genetisch bedingten Tumorerkrankung.

In zahlreichen Gesprächen mit MEN2-Familien wurde immer wieder berichtet, dass in den Vorgenerationen Familienangehörige bereits in mittleren Lebensjahren am plötzlichen Herztod verstorben seien – Folgen einer seinerzeit unerkannten und weit fortgeschrittenen syndromalen PCC-Erkrankung, mit und ohne zeitgleich bestehende klinisch manifeste Schilddrüsenkarzinomerkrankung. Mit der molekulargenetischen Identifikation der Genmutationsträger wurde beides möglich: die Früherkennung und -behandlung des MTC und des PCC. Die heute allgemein akzeptierte adrenokortikal-schonende subtotale Adrenalektomie schafft darüber hinaus, im Gegensatz zu der früher routinemäßig vorgenommenen totalen Adrenalektomie, beste Voraussetzungen für ein kortisonsubstitutionsfreies selbstbestimmtes Leben [87].

Nach den ersten Erfahrungen mit der DNA-basierten prophylaktischen Thyreoidektomie [4–7] und dem Nachweis der Genotyp-Phänotyp-Korrelation [77–79] kam es darauf an, dass chirurgische Konzept dergestalt zu präzisieren, dass mit dem individuell besten Zeitpunkt der prophylaktischen Thyreoidektomie der kritische, prognostisch entscheidende Schritt der C‑Zell-Transformation vom nodal-negativen zum nodal-positiven MTC nicht verpasst wurde. Es bedurfte 15 Jahre intensiver klinischer Forschung, bis 2009 anhand eines ausreichend großen Krankengutes der „Kipp-Punkt“ der Tumorerkrankung mit dem Überschreiten des oberen Kalzitoninreferenzwertes festgelegt wurde [55, 71]. Dadurch konnte die zunächst allein mutationsorientierte Altersempfehlung für die Durchführung der prophylaktischen Thyreoidektomie bei asymptomatischen Genmutationsträgern zugunsten einer mutations- und altersunabhängigen kalzitoninbasierten Zeitpunktempfehlung abgelöst werden. Die Altersempfehlung spiegelte den C‑Zell-Transformationsprozess zu ungenau wieder, um den Eltern genmutationstragender Kinder einen verlässlichen, hinreichend präzisen und planbaren Anhaltspunkt für das Timing des Schilddrüseneingriffs bei ihren Kindern zu geben. Die Kalzitoninbestimmung eröffnet ihnen nun die Wahl zwischen einer „Last-minute“-Thyreoidektomie oder einer „frühzeitig prophylaktischen“ Operation bei noch stabilen oder nur langsam innerhalb des Normbereiches ansteigenden Kalzitoninwerten.

Ein weiterer entscheidender Schritt zur Verbesserung des onkologischen Outcomes speziell bei der aggressivsten Form der MEN2-Erkrankung, dem in 95 % „de novo“ auftretenden MEN2B-Syndroms, gelang durch eingehende Befragungen der Eltern von MEN2B-Kindern. Hierbei zeigte sich, wie 2004 und 2008 erstmals beschrieben, dass nicht die biochemischen oder klinischen Symptome der Hyperkalzitoninämie eine frühzeitige Diagnose und Therapie der MEN2B ermöglichen, sondern nichtendokrine Symptome, die zunächst keinen nosologischen Zusammenhang mit der genetisch basierten C‑Zell-Erkrankung vermuten ließen: das tränenlose Weinen und die Obstipation der Neugeborenen [75, 76]. Das onkologische Outcome der MEN2B-Genträger kann durch frühzeitige Erkennung dieser nichtendokrinen Symptome entscheidend verbessert werden [72]. Wenn heute MEN2B-Eltern die Gentestung bei ihrem mutationspositiven Kind zeitnah nach der Geburt vornehmen lassen, kann durch prophylaktische Thyreoidektomie mit optimalem Operationszeitpunkt im 2. Lebenshalbjahr in fast allen Fällen die C‑Zell-Erkrankung geheilt werden [74].

Die Kalzitoninwerte sind im 1. bis 3. Lebensjahr bereits physiologisch erhöht, was eine präzise kalzitoninbasierte Operationsstrategie hinsichtlich der Notwendigkeit einer zentralen oder kombiniert zentralen und lateralen Kompartmentresektion erschwert. In der von Brauckhoff und Mitarbeitern publizierten Studie [72] hatten die biochemisch geheilten MEN2B-Kinder im Alter von 0,5 bis 3,7 Jahren basale Kalzitoninwerte zwischen 27 pg/ml (oberer Assay-Normbereich für weibliche Testpersonen < 5 pg/ml) und 105 pg/ml (oberer Assay-Normbereich für männliche Testpersonen < 10 pg/ml). Aus einer kürzlich von Machens und Mitarbeitern publizierten Studie [24] ergibt sich hinsichtlich der Frage des Ausmaßes der lateralen Kompartmentresektion, dass sich nicht nur beim sporadischen MTC [19, 20, 22, 23], sondern auch beim hereditären, hier am MEN2B-untersuchten MTC, die Möglichkeit ergibt, die intraoperative Desmoplasiehistologie zur Entscheidungsfindung hinzuzuziehen. Bei desmoplasienegativen MEN2B-MTC waren die ipsilateral lateralen Lymphknoten im Gegensatz zu den desmoplasiepositiven MEN2B-MTC nicht tumorbefallen. Die bislang nur an wenigen Fällen erzielten Ergebnisse bedürfen weiterer Untersuchungen, um daraus belastbare chirurgische Strategien abzuleiten, zeigen jedoch einen Weg auf, das Resektionsausmaß besser präzisieren und ggf. einschränken zu können. Dies gilt insbesondere für MEN2B-Patienten, bei denen im 1. Lebensjahr die Kalzitoninwerte bereits physiologisch erhöht sind, aber auch für MEN2A-Patienten mit MTC.

Es existieren derzeit keine deutschlandweiten, populationsbezogenen Daten zur Effektivität routinemäßiger Kalzitoninbestimmungen bei Patienten mit Knotenstruma, zum Familienscreening bei Indexpatienten oder zur Häufigkeit prophylaktischer Thyreoidektomien bei normokalzitoninämischen Genmutationsträgern. Aus den vorliegenden Untersuchungen ist jedoch abzuleiten, dass es nach Einleitung der molekulargenetischen Ära nicht nur zu einem „Gestaltwandel“ des hereditären MTC gekommen ist, sondern parallel hierzu auch zu einer beeindruckenden Verbesserung des Outcomes der Betroffenen [9, 94]. Unter Berücksichtigung eines potenziellen Selektionsbias verringerte sich im eigenen Krankengut die Häufigkeit von Indexpatienten einer MEN2A-Erkrankung schrittweise von insgesamt 41–74 % auf null, diejenige mit Nachweis eines MTC von 96–100 % auf 33 % bis auf null und die Heilungsraten der Träger von High-risk- und Low-moderate-Mutationen stiegen von 17–33 % auf 100 % an [9]. Auch der Alterszeitpunkt der PCC-Operationen verlagerte sich zu jüngeren Altersgruppen, sodass das Konzept der funktionserhaltenden Adrenalektomie [87] zunehmend umgesetzt werden konnte.

Mit dem Konzept der prophylaktischen Thyreoidektomie ist verbunden, dass ebenso wie Indikation und Zeitpunkt der Operation auch die Nachsorge des operierten Kindes bzw. Jugendlichen sehr eingehend bereits bei der Operationsplanung besprochen werden muss. Dies betrifft insbesondere die altersgerechte Schilddrüsenhormonsubstitution und deren Kontrolle. In einem von Frank-Raue untersuchten Kollektiv von 46 prophylaktisch thyreoidektomierten Kindern und Jugendlichen zeigte sich im Rahmen der Nachsorge bei ca. einem Drittel von ihnen zwar keine klinisch erkennbare Hypothyreose, jedoch ein z. T. erheblich erhöhtes TSH [95]. Die Bereitschaft prophylaktisch thyreoidektomierter Patienten, die postoperativ erforderliche Substitution mit Levothyroxin gemäß Medikamentenplan korrekt durchzuführen, ist in den meisten Fällen gegeben, weil ja bereits ein Elternteil postoperativ ebenfalls substitutionspflichtig ist. Hierbei kann es dennoch, wie diese Untersuchung zeigte, zu Compliance-Problemen kommen. Eine sorgsame, sowohl altersgerechte als auch den individuellen Genotyp und Phänotyp berücksichtigende Nachsorge ist daher integrativer Bestandteil des Konzepts der prophylaktischen Thyreoidektomie.

Das komplikative Risiko der prophylaktischen Thyreoidektomie bestimmt weniger das Alter der operierten Kinder und Jugendlichen, bei denen im eigenen Krankengut kein Unterschied in den einzelnen Altersgruppen von < 3 Jahren bis 18 Jahren bestand [90], als vielmehr die ggf. zeitgleich notwendige Lymphknotendissektion des zentralen Halskompartments. Hierbei waren passagere Komplikationen wie postoperative Hypokalzämie und Stimmlippenparese führend, dauerhafte Komplikationen ergaben sich im eigenen Krankengut jedoch nicht. In jedem Fall ist das durch zentrale Lymphknotendissektion erhöhte komplikative Risiko ein starkes Argument für die Durchführung der prophylaktischen Thyreoidektomie im Stadium der Normokalzitoninämie, die eine Lymphknotendissektion entbehrlich macht.

## Synopsis und Ausblick

**Abb. 6:**
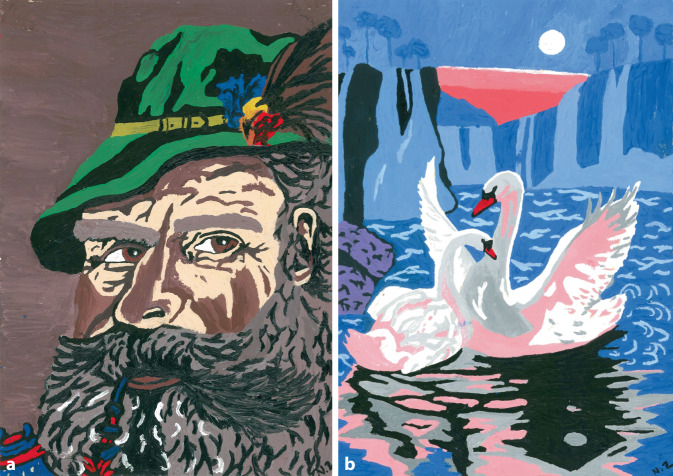
Amateurzeichnungen einer 17-jährigen MEN2B-Patientin **a** vor und **b** nach Operation ihres bereits lokal fortgeschrittenen MTC

30 Jahre genmutation- und kalzitoninbasierte prophylaktische Thyreoidektomie beim MEN2-Syndrom: ein Meilenstein und beeindruckendes Beispiel translationaler Medizin „from bedside to bench to bedside“ usw. (Tab. 2). Beeindruckend, weil intensive Kooperation auf nationaler und internationaler Ebene eine Frühdiagnose des MEN2-Syndroms schon vor Erkrankungsbeginn bzw. im klinisch noch asymptomatischen Krankheitsstadium eröffnet hat. Diese Frühdiagnose erlaubt wiederum eine individuelle Präzisierung des im Einzelfall erforderlichen Resektionsmindestausmaßes. So konnten selbst bei der aggressivsten Form des MEN2B-MTC die onkologischen Behandlungsergebnisse grundlegend verbessert werden. Einziger Wermutstropfen ist der nach wie vor unumgängliche Verlust der Schilddrüse mit der Notwendigkeit lebenslanger medikamentöser Substitution. Des Weiteren konnte die Lebensqualität der Zweiterkrankung des PCC durch in einem früheren Stadium durchgeführte Resektionsverfahren mit Erhalt größerer Anteile tumorfreien Nebennierengewebes deutlich verbessert und dadurch die früher nach bilateral totaler Adrenalektomie auftretenden Addison-Krisen [96] vermieden werden. Die Furcht vor der Erkrankung (Abb. 6) ist für die meisten Betroffenen berechtigter Hoffnung auf Heilung gewichen. Inwieweit durch zukünftige Gentechniken das Erkrankungsrisiko vollständig eliminiert werden kann, bleibt abzuwarten.

**Tab. 2:** Geschichte und Entwicklung der Erforschung des hereditären MTC: Meilensteine einer syndromalen Erkrankung in Diagnostik und Therapie (Auswahl)

Jahr	Meilenstein
1876	Vermutliche Erstbeschreibung der C‑Zellen beim Hund [27]
1901	Vermutliche Erstbeschreibung eines MEN2B-MTC [15]
1926	Erste klinisch-pathologische Klassifikation der Schilddrüsentumoren und des MTC [17]
1959	Erste Definition des MTC als klinisch-pathologische Entität [16]
1961/1964	Erstbeschreibungen des thyreoidalen Kalzitonins [28, 29]
1961–1968	Erstbeschreibungen des MEN2-Syndroms [39–44]
1974	Erste WHO-Klassifikation der Schilddrüsentumoren mit MTC als eigenständige Entität sporadischer und familiärer MTC [25]
1993/1994	Erstbeschreibungen des molekularen MEN2-Genlokus auf dem *RET*-Protoonkogen [1–3]
1994–1998	Erstbeschreibungen der DNA-basierten prophylaktischen Thyreoidektomie [4, 5, 7]
1996–2003	Genotyp-Phänotyp-Korrelation zur Risikostratifizierung des hereditären MTC [77–79]
1994–2004	Implementierung der routinemäßigen Kalzitoninbestimmung bei Patienten mit Knotenstruma [59, 80]
2004/2008	Erstbeschreibungen der nichtendokrinen Frühsymptome des MEN2B-Syndroms [75, 76]
2009	Individualisierung des Zeitpunktes der prophylaktischen Thyreoidektomie durch Kalzitoninbestimmung bei asymptomatischen Genmutationsträgern [55]
2024	Erstbeschreibung der desmoplasieabhängigen Lymphknotenmetastasierung beim hereditären MTC [24]
